# Skull Base Osteomyelitis Complicated by Internal Carotid Artery Thrombosis and Sigmoid Sinus Thrombosis

**DOI:** 10.7759/cureus.111204

**Published:** 2026-06-20

**Authors:** Zakaria Toufga, Aymane El Farouki, Meriem Edderai, Rachida Saouab, Hassan En Nouali

**Affiliations:** 1 Department of Radiology, Mohammed V Military Hospital, Mohammed V University, Rabat, MAR

**Keywords:** diabetes mellitus, internal carotid artery thrombosis, mri, otomastoiditis, sigmoid sinus thrombosis, skull base osteomyelitis, staphylococcus

## Abstract

Skull base osteomyelitis (SBO) is a rare and potentially fatal deep-space infection most commonly arising from malignant external otitis in immunocompromised or diabetic patients. Vascular complications, including septic thrombosis of the internal carotid artery (ICA) and dural venous sinuses, are among the most severe and life-threatening sequelae, yet remain underreported in the radiological literature. We present the case of a 59-year-old male with type 2 diabetes mellitus who initially presented with severe, refractory left-sided otalgia, purulent otorrhea, and progressive lower cranial nerve palsies. He was diagnosed with left-sided SBO on MRI, characterised by extensive infiltration of the carotid, lateral pharyngeal, and perivertebral spaces, with osseous involvement of the clivus, left occipital condyle, the anterior arch of C1, and the left mastoid. Critically, MRI demonstrated encasement and non-opacification of the cervical segment of the left internal carotid artery, thrombosis of the left internal jugular vein with extension to the left sigmoid sinus, and ipsilateral otomastoiditis. This case illustrates the pivotal diagnostic value of multiparametric MRI, combining fluid-attenuated inversion recovery, diffusion-weighted, and post-gadolinium sequences, in characterising the full extent of SBO and its neurovascular complications, findings that are critical for guiding urgent multidisciplinary management.

## Introduction

Skull base osteomyelitis (SBO) is a severe, potentially life-threatening infection of the bony structures of the skull base, most frequently arising as a complication of malignant (necrotising) external otitis. It predominantly affects elderly patients with diabetes mellitus or other immunocompromising conditions, with Pseudomonas aeruginosa classically identified as the principal causative organism [1,2]. However, non-pseudomonal aetiologies, including Staphylococcus aureus, are increasingly recognised, particularly in cases with a haematogenous mechanism or an atypical clinical presentation [3].

The clinical presentation of SBO is often insidious, encompassing otalgia, headaches, and cranial nerve deficits, which may lead to diagnostic delay. Among the most feared complications of SBO are vascular injuries, including septic thrombosis of the internal carotid artery (ICA) and sigmoid sinus, both of which carry a high risk of stroke, intracranial hypertension, and death [4,5]. These complications are best characterised by MRI, which provides superior soft-tissue contrast and multiparametric capabilities compared with computed tomography [6].

We herein report a case of SBO in a 59-year-old diabetic male in whom MRI revealed an exceptionally extensive disease, including thrombosis of the cervical ICA and the left sigmoid sinus, highlighting the critical role of comprehensive MRI evaluation in guiding urgent management decisions.

## Case presentation

A 59-year-old male with a known history of type 2 diabetes mellitus, managed with oral hypoglycaemic agents, presented with severe, refractory left-sided otalgia, purulent otorrhea, and progressive lower cranial nerve palsies, along with a two-week history of progressively worsening headaches, left-sided facial pain, and diplopia. On admission, the patient was afebrile, haemodynamically stable, with poorly controlled glycaemia (fasting blood glucose: 16.2 mmol/L; HbA1c: 9.8%). Neurological examination revealed a left abducens nerve palsy and hypoaesthesia in the territory of the left trigeminal nerve. There was no cervical lymphadenopathy or palpable neck mass.

Laboratory investigations showed an elevated C-reactive protein (CRP: 118 mg/L; reference <5 mg/L), raised erythrocyte sedimentation rate (ESR: 82 mm/h; reference range: <15 mm/h for men/<20 mm/h for women), and leukocytosis (14.1 × 10⁹/L; reference range: 4.0−10.0×10^9^/L). Blood cultures yielded Staphylococcus aureus (methicillin-sensitive; MSSA), while HIV serology was negative. Nasopharyngoscopy revealed no mucosal lesion or mass in the nasopharynx.

MRI demonstrated extensive fluid-attenuated inversion recovery (FLAIR) hyperintense infiltration involving the left carotid space, the left lateral pharyngeal space, and the left perivertebral space, with partial extension into the left posterior cervical space (Figure 1A). This infiltration showed restricted diffusion on DWI sequences (Figure 1B) and avid gadolinium enhancement (Figure 1C), consistent with active phlegmonous-infectious involvement. Heterogeneous T1 and T2 signal abnormalities were identified within the clivus, the left occipital condyle, the anterior arch of the left C1 vertebra, and the left mastoid, all demonstrating enhancement after gadolinium administration (Figure 1D), in keeping with multisite SBO.

**Figure 1:**
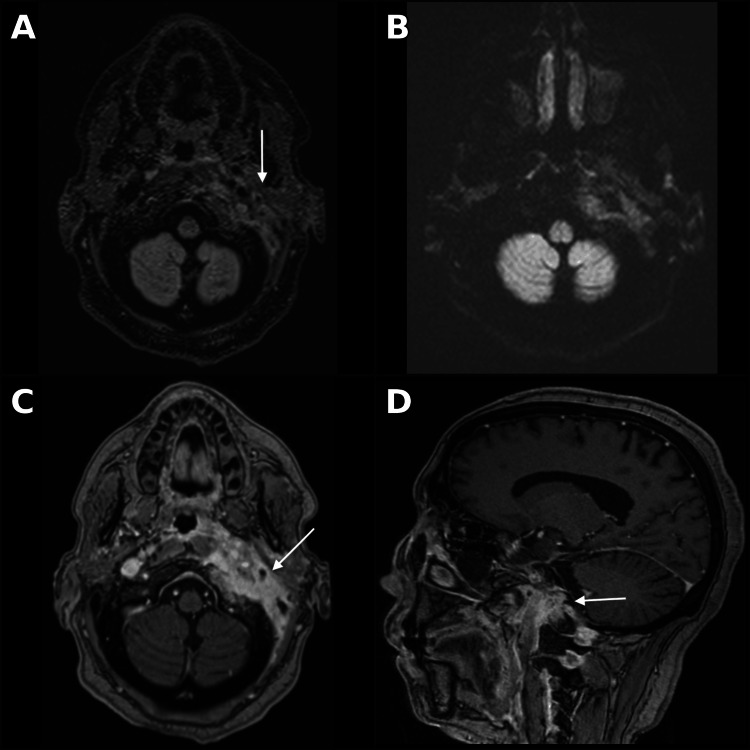
Multiparametric MRI demonstrating left-sided skull base osteomyelitis. (A) Axial FLAIR sequence showing hyperintense infiltration of the left carotid space and lateral pharyngeal space, with extension to the left perivertebral space (arrow). (B) Axial diffusion-weighted image (DWI) demonstrating restricted diffusion within the same infiltrated spaces, consistent with active infectious phlegmon. (C) Axial post-gadolinium T1-weighted image showing avid and heterogeneous enhancement of the skull base and the surrounding deep cervical spaces, with encasement of the left internal carotid artery (arrow). (D) Sagittal post-gadolinium T1-weighted image demonstrating extensive enhancement of the clivus, the anterior arch of C1, and the left occipital condyle (arrow), consistent with multisite skull base osteomyelitis. FLAIR: Fluid-attenuated inversion recovery

Critically, the infectious infiltration was seen to encase the left ICA, with complete absence of flow signal in the cervical segment of the left ICA on post-contrast sequences, indicating ICA thrombosis (Figure 2A). The left internal jugular vein was thrombosed, with propagation of thrombosis into the left sigmoid sinus (Figure 2B). 

**Figure 2:**
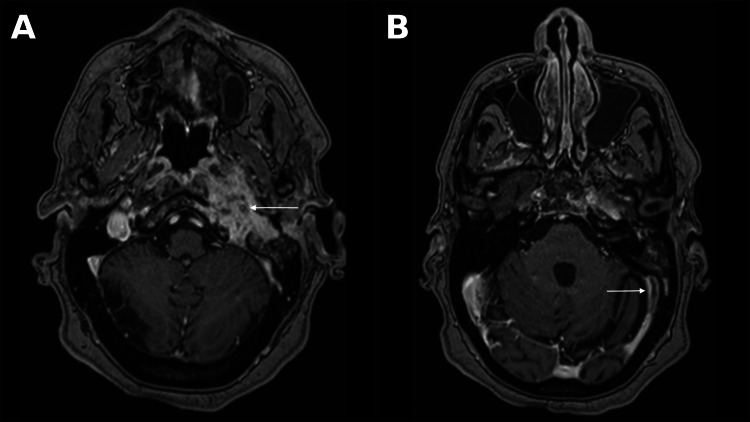
Vascular complications of skull base osteomyelitis on MRI. (A) Post-gadolinium T1-weighted axial image showing absence of opacification of the cervical segment of the left internal carotid artery (arrow), consistent with septic ICA thrombosis; note the surrounding enhancing infectious infiltration. (B) Axial post-contrast T1 image at the level of the posterior fossa demonstrating lack of enhancement of the left sigmoid sinus (arrow), in keeping with sigmoid sinus thrombosis. ICA: Internal carotid artery

The overall MRI findings were diagnostic of left-sided SBO with associated thrombosis of the cervical left ICA and left internal jugular vein extending to the left sigmoid sinus.

Following multidisciplinary discussion with infectious disease, neurology, neurosurgery, and otolaryngology specialists, the patient was commenced on intravenous antistaphylococcal penicillins (oxacillin) combined with ciprofloxacin and therapeutic anticoagulation with low-molecular-weight heparin (enoxaparin) for the ICA and sigmoid sinus thromboses. Glycaemic optimisation was instituted via insulin infusion. Clinical improvement, with reduction in headache severity and partial recovery of the abducens palsy, was observed over three weeks of inpatient treatment. CRP declined progressively to 14 mg/L by day 28. The patient was discharged on oral fluoroquinolones (ciprofloxacin) combined with rifampin and anticoagulation with direct oral anticoagulants (rivaroxaban) with a planned 12-week follow-up.

## Discussion

Our case illustrates an exceptionally severe and radiologically rich presentation of SBO, notable for the simultaneous occurrence of septic ICA thrombosis, sigmoid sinus thrombosis, and otomastoiditis in a diabetic patient with a Staphylococcal bacteraemia. This extensive neurovascular involvement correlated directly with the patient's critical clinical presentation, which was dominated by debilitating hemicranial headache, severe otalgia, and a progressive cranial nerve palsy. Each of these features warrants individual discussion.

The deep cervical space involvement observed in this case, encompassing the carotid space, lateral pharyngeal space, and perivertebral space with extension to the posterior cervical space, reflects the propensity of SBO to spread along fascial planes of least resistance. The skull base communicates freely with these cervical compartments through anatomical foramina, and once infection transgresses the petrous bone or the clivus, contiguous spread into the deep cervical spaces is well recognised [2,7]. The FLAIR and T2 hyperintensity with restricted diffusion and avid gadolinium enhancement observed in our patient provided a comprehensive characterisation of both the infectious phlegmon and the osseous involvement across multiple skull base structures [6,8].

The thrombosis of the cervical segment of the left ICA is among the most alarming complications documented in this case. Septic ICA thrombosis in the context of SBO is rare but carries a high risk of ischaemic stroke and death [4,5]. It results from direct inflammatory encasement of the artery within the carotid space, leading to endothelial injury, luminal thrombosis, and, in severe cases, pseudoaneurysm formation. On MRI, the absence of the normal intraluminal flow void on T2 sequences and the lack of enhancement on post-gadolinium imaging are the key diagnostic indicators of ICA occlusion, as observed in our patient [9]. Prompt recognition of this complication is essential, as it may necessitate urgent neurosurgical or endovascular intervention.

Concomitant thrombosis of the internal jugular vein with sigmoid sinus extension represents a further life-threatening vascular complication. Sigmoid sinus thrombosis in SBO typically arises from direct contiguous spread from the adjacent mastoid and posterior petrous bone, a pathway clearly illustrated by the ipsilateral otomastoiditis in our patient [10]. MRI, particularly post-gadolinium T1-weighted sequences, demonstrated the characteristic absence of sinus enhancement (“empty delta sign” equivalent in the sigmoid sinus), confirming thrombosis. This finding prompted the addition of therapeutic anticoagulation to the management regimen, a decision that would not have been possible without comprehensive MRI assessment.

From a microbiological standpoint, the identification of Staphylococcus aureus (MSSA) as the causative organism is noteworthy. Although Pseudomonas aeruginosa remains the classic pathogen in otogenic SBO, Staphylococcal SBO is increasingly documented in the literature, particularly in non-otogenic cases or those with associated bacteraemia [3]. The absence of classical otalgia or otorrhoea in our patient, combined with MSSA bacteraemia, suggests a haematogenous route of infection, a mechanism facilitated by the impaired innate immunity characteristic of poorly controlled diabetes mellitus.

The normalised nasopharyngeal mucosa and absence of a nasopharyngeal mass on both MRI and nasopharyngoscopy are important negative findings in this case. Nasopharyngeal carcinoma (NPC) is a critical differential diagnosis for any patient presenting with skull base destruction and cranial nerve palsies, particularly in regions of high prevalence. The preservation of mucosal relief and the absence of a soft-tissue mass, combined with the clinical and biological infectious context, supported the diagnosis of SBO and effectively excluded NPC as an alternative aetiology [2,8].

This case reaffirms the superior diagnostic value of multiparametric MRI in SBO. CT, whilst useful for detecting cortical bone destruction, is limited in its ability to characterise soft-tissue extension, vascular involvement, and early marrow infiltration. MRI, by combining T2, FLAIR, DWI, and post-gadolinium sequences, provides a comprehensive, one-stop assessment of disease extent and complications, directly informing multidisciplinary management decisions [6,8].

## Conclusions

This case demonstrates that SBO can present with devastating vascular complications, including simultaneous ICA and sigmoid sinus thrombosis, which carry a high risk of ischaemic stroke and intracranial hypertension. Multiparametric MRI is indispensable in this setting, enabling accurate diagnosis, complete mapping of disease extent, and identification of life-threatening neurovascular complications that directly guide urgent therapeutic decisions. Radiologists must be vigilant for the full spectrum of SBO complications, and nasopharyngeal carcinoma must always be systematically excluded in any patient presenting with skull base involvement and cranial nerve deficits.
